# Audio-Visual Temporal Recalibration Can be Constrained by Content Cues Regardless of Spatial Overlap

**DOI:** 10.3389/fpsyg.2013.00189

**Published:** 2013-04-24

**Authors:** Warrick Roseboom, Takahiro Kawabe, Shin’Ya Nishida

**Affiliations:** ^1^Human Information Science Laboratory, NTT Communication Science LaboratoriesAtsugi, Japan

**Keywords:** lag adaptation, temporal recalibration, audio-visual, multisensory, speech perception, spatial, contextual

## Abstract

It has now been well established that the point of subjective synchrony for audio and visual events can be shifted following exposure to asynchronous audio-visual presentations, an effect often referred to as temporal recalibration. Recently it was further demonstrated that it is possible to concurrently maintain two such recalibrated estimates of audio-visual temporal synchrony. However, it remains unclear precisely what defines a given audio-visual pair such that it is possible to maintain a temporal relationship distinct from other pairs. It has been suggested that spatial separation of the different audio-visual pairs is necessary to achieve multiple distinct audio-visual synchrony estimates. Here we investigated if this is necessarily true. Specifically, we examined whether it is possible to obtain two distinct temporal recalibrations for stimuli that differed *only* in featural content. Using both complex (audio visual speech; see Experiment 1) and simple stimuli (high and low pitch audio matched with either vertically or horizontally oriented Gabors; see Experiment 2) we found concurrent, and opposite, recalibrations despite there being no spatial difference in presentation location at any point throughout the experiment. This result supports the notion that the content of an audio-visual pair alone can be used to constrain distinct audio-visual synchrony estimates regardless of spatial overlap.

## Introduction

Many events in our everyday environment produce signals that can be perceived by multiple sensory modalities. For example, human speech produces correlated signals in both visual and auditory modalities. Critically, the information perceived by different sensory modalities is initially processed independently and subsequently combined to form a coherent percept. When the sources are redundant, the accuracy of perceptual judgments can be enhanced (Stein and Meredith, 1993; Ernst and Banks, 2002; Alais and Burr, 2004; Arnold et al., 2010). However, a challenge to this process is that a common source of origin for two sensory signals does not guarantee a common perception of time due to differences in both extrinsic and intrinsic signal speeds (Spence and Squire, 2003; King, 2005). With regards to audio and visual signals, sound (∼330 m/s) travels through air more slowly than light (∼300,000,000 m/s). After reaching sensory receptors, transduction of sound by the hair cells of the inner ear is quicker than photo-transduction of light by the retina, resulting in processing latency differences up to ∼50 ms (King, 2005). These differences in physical and neural transmission speeds will cancel each other out at observer distances of ∼10–15 m, but stimulus attributes can also contribute to this variance. For example, speed of neural propagation is correlated with signal intensity (Roufs, 1963; Lennie, 1981; Williams and Lit, 1983; Burr and Corsale, 2001; Kopinska and Harris, 2004). By a related means, attention also likely contributes (e.g., prior entry; Titchener, 1908; Spence et al., 2001). Consequently, discrepancies in the relative timing of audio and visual signals in the order of 10’s of milliseconds can be expected at varying event distances and signal intensities.

As our perception of nearby audio-visual events typically contains minimal apparent temporal discrepancy, a critical question regards what possible processes the brain may utilize to create such coherent perception. It has recently been proposed that one strategy to overcome the problem of differential transmission speeds would be to dynamically calibrate audio-visual timing perception based on recent events (Fujisaki et al., 2004; Vroomen et al., 2004; Heron et al., 2007). In support of this idea, many studies (e.g., Fujisaki et al., 2004; Vroomen et al., 2004; Navarra et al., 2005, 2009, 2012; Miyazaki et al., 2006; Heron et al., 2007, 2010, 2012; Keetels and Vroomen, 2007; Vatakis et al., 2007, 2008; Hanson et al., 2008; Harrar and Harris, 2008; Di Luca et al., 2009; Roach et al., 2011; Roseboom and Arnold, 2011; Tanaka et al., 2011; Yarrow et al., 2011a,b; Machulla et al., 2012; Yuan et al., 2012; see Vroomen and Keetels, 2010 for review) have demonstrated that following exposure (adaptation) to a short period (<∼3 mins) containing repeated presentations of audio-visual pairs in which the audio and visual components are presented asynchronously (∼100–300 ms), observers’ point of subjective synchrony (PSS) between audio and visual events shifts in the direction of the exposed asynchrony (i.e., observers report physical offsets between audio and visual events in the exposed direction, for example audition lagging vision, as synchronous more often than they had prior to the exposure period). This change is sometimes accompanied by a change in the width of the response distribution (reported either by the just noticeable difference; JND, standard deviation; SD, or full-width half-maximum; FWHM of the distribution) such that observers respond with less temporal precision following adaptation to asynchrony.

Subsequent studies support the existence of similar recalibration processes for many different combinations of both multisensory (Navarra et al., 2007; Hanson et al., 2008; Harrar and Harris, 2008; Di Luca et al., 2009) and unisensory signal pairs (Bennett and Westheimer, 1985; Okada and Kashino, 2003; Arnold and Yarrow, 2011). These results suggest that sensory recalibration occurs supra-modally. Combined with results demonstrating that temporal recalibration may transfer across stimuli or tasks (Fujisaki et al., 2004; Keetels and Vroomen, 2007; Di Luca et al., 2009; Navarra et al., 2009, 2012), these studies indicate that sensory recalibration may represent a change in a generalized mechanism of timing perception. However, humans exist in a spatio-temporally cluttered world with the possibility of perceiving one or more multisensory events, each at a different distance and with differing signal intensities, in close temporal succession. In such an environment, maintaining a single estimate of synchrony generalized across all possible event pairs may not be beneficial for facilitating accurate perception of any given signal pair. Accordingly, it might be possible that humans can concurrently maintain multiple, distinct, estimates of audio-visual synchrony. The results of two recent studies (Roseboom and Arnold, 2011; Heron et al., 2012) support such a premise.

A study by Roseboom and Arnold (2011) utilized male and female audio-visual speech stimuli and demonstrated that it is possible for observers to concurrently maintain two temporally opposing estimates of audio-visual synchrony. For example, one estimate for the female identity where audition preferably leads vision, and one estimate for the male identity where audition preferably lags vision. A subsequent study by Heron et al. (2012) replicated this finding for simple stimuli, and further suggested that the spatial location, not the content of stimuli, might constrain differential temporal recalibrations. Using pairs of high or low spatial frequency Gabor’s paired with high or low temporal frequency auditory tones they presented all stimuli from the same physical location. This configuration revealed no evidence for differential temporal recalibrations dependent on the content of the stimuli. However, when presenting two identical audio and visual stimuli (Gaussian luminance blobs and auditory white noise) from different spatial locations (left or right of fixation with matched auditory location), the results clearly demonstrated opposite temporal recalibrations constrained by the physical presentation location. This result was consistent with the spatial specificity often shown by temporal adaptation effects (Johnston et al., 2006; Ayhan et al., 2009; Bruno et al., 2010).

However, the result is apparently inconsistent with that reported by Roseboom and Arnold (2011). In this study it was revealed that the recalibrated synchrony estimates for a given stimulus identity (male or female) did not change whether the stimuli were presented from the same or different spatial location from that in which they were presented during the adaptation period. This result indicated that the differential recalibrations were constrained not by the spatial position of presentation but were contingent primarily on the content of the stimulus, in this case the identity of the speaker (i.e., male or female). This suggestion is broadly consistent with several other recent results demonstrating that temporal perception of audio-visual displays can be modulated by the content or featural relation of the signals (e.g., Vatakis and Spence, 2007; Parise and Spence, 2009; Roseboom et al., 2013).

In trying to reconcile this difference, Heron et al. (2012) pointed to the fact that the stimuli in Roseboom and Arnold (2011) reliably differed during the adaptation phase not only in content (identity) but also in visual spatial location of presentation. By comparison, in Heron et al. (2012) investigation of content constrained temporal recalibration the stimuli were only ever presented from a single central location. One might take this to imply that spatial dissociation, at least during the initial adaptation sequence, may be a critical factor for determining the appropriate audio-visual correspondences in order for a content constrained recalibration to be revealed. However, an alternative interpretation is that while difference in spatial location is an effective factor to facilitate audio-visual correspondence during adaptation, other factors such as featural or content difference may also be able to play a similar role. According to this idea, a spatial location difference is not absolutely necessary to produce differential temporal recalibrations – featural difference may be sufficient.

The role of spatial specificity in temporal recalibration is a critical question. Close spatio-temporal correspondence has been demonstrated to be a critical feature for the most basic level of multisensory integration in the mammalian brain (see Stein and Meredith, 1993). While featural correspondence has not been demonstrated to play such a fundamental role in multisensory perception, an array of different natural featural correspondences between different audio and visual pairs have been demonstrated (e.g., high temporal frequency sounds and high spatial frequency visual gratings; Evans and Treisman, 2010). However, the evidence to suggest that these correspondences are anything more than common decisional strategies is controversial (see Spence and Deroy, 2013 for a recent review). Consequently, a characterization of temporal recalibration as a general process, utilizing information from many dimensions of event difference, including spatial, temporal, and featural correspondence, implies different processing requirements to a more specified process constrained only by spatio-temporal relation. We were interested in determining why the results of Roseboom and Arnold (2011) and Heron et al. (2012) support such different characterizations. We wanted to know if it was possible to obtain equivalent results to those reported by Roseboom and Arnold (2011) in stimulus displays that contain no spatial disparity during either the adaptation or test phases.

## Experiment 1

**Figure 1:**
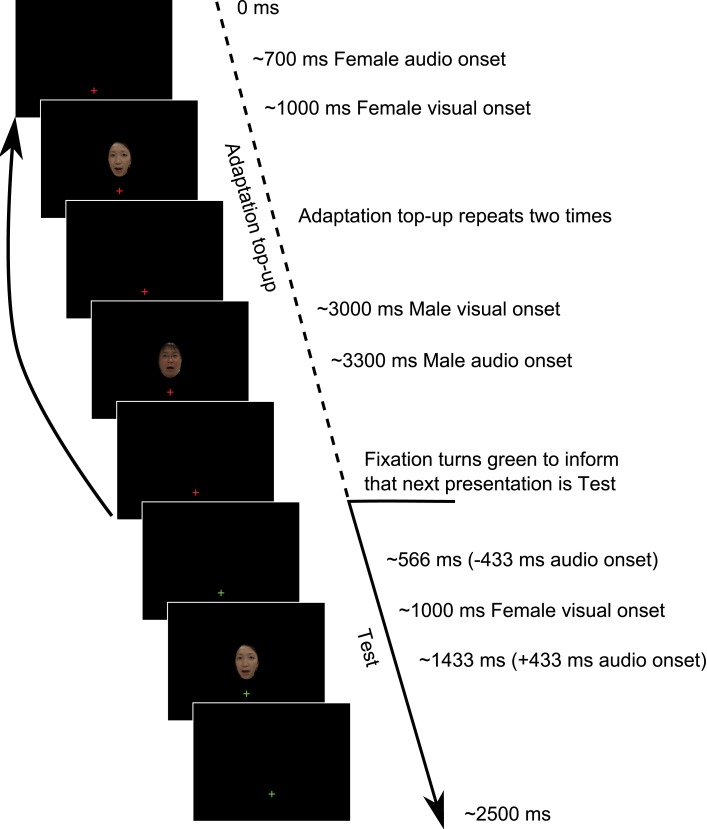
**Example Test trial sequence from Experiment 1**. Each trial began with an Adaptation top-up period in which two repeats of each of the adapting relationships from the previously presented Adaptation phase were repeated. Following the top-up period, participants were informed by a change in the fixation cross from red to green that the next presentation would be a Test presentation to which they would have to respond. The Adaptation phase consisted of 40 repeats of each stimulus configuration, as depicted in the Adaptation top-up period, before proceeding onto the Adaptation top-up/Test presentation cycle.

In the first experiment we constructed a paradigm similar to that previously used by Roseboom and Arnold (2011), with some minor differences. The stimuli were male or female actors saying “ba” (see Figure 1; Movie S1 in Supplementary Material for example). Critically, there was no difference in spatial location of presentation for the different identity stimuli during any phase of the experiment. As such, this experiment was designed to explicitly confirm whether it is necessary to have spatial disparity during the adaptation stage of the experiment to obtain multiple, concurrent, audio-visual temporal recalibrations constrained only by featural differences for audio-visual speech stimuli.

### Participants

There were eight participants, all naïve as to the experimental purpose. All reported normal or corrected to normal vision and hearing. Participants received ¥1000 per hour for their participation. Ethical approval for this study was obtained from the ethical committee at Nippon Telegraph and Telephone Corporation (NTT Communication Science Laboratories Ethical Committee). The experiments were conducted according to the principles laid down in the Helsinki Declaration. Written informed consent was obtained from all participants.

### Apparatus and stimulus

Visual stimuli were generated using a VSG 2/3 from Cambridge Research Systems (CRS) and displayed on a 21″ Sony Trinitron GDM-F520 monitor (resolution of 800 × 600 pixels and refresh rate of 120 Hz). Participants viewed stimuli from a distance of ∼57 cm. Audio signals were presented binaurally via Sennheiser HDA200 headphones. Audio stimulus presentations were controlled by a TDT RM1 Mobile Processor (Tucker-Davis Technologies). Auditory presentation timing was driven via a digital line from a VSG Break-out box (CRS), connected to the VSG, which triggered the RM1. Participants responded using a CRS CT3 response box.

The stimuli consisted of 500 ms movies of native Japanese speakers, either male or female, saying “ba” (recorded using a Sony Handycam HDR-CX560). The visual components of these recordings were sampled at a rate of 30 frames per second. Visual stimuli were presented within an oval aperture (5.65° of visual angle wide, 7.65° of visual angle high) centered 5.75° of visual angle above a central fixation cross (which subtended 0.6° of visual angle in width and height) against a black background (see Figure 1; Movie S1 in Supplementary Material for depiction). Auditory signals were produced from the original movies (16 bit sample size, mono) and were normalized to a peak sound intensity of ∼65 db SPL. A “Hiss and Hum” filter was applied to audio stimuli below 20 db (using WavePad Audio Editor, NCH Software).

The experiment consisted of two phases, Adaptation and post-adaptation Test. During the Adaptation phase participants observed 40 presentations of each of the male and female stimuli, sequentially alternating between the two (see Movie S1 in Supplementary Material for example trial sequence). The two audio-visual stimuli possessed opposite audio-visual temporal relationships, such that, for example (as in Figure 1; Movie S1 in Supplementary Material), the onset of the audio stream of the female voice occurred *prior* to the onset of the female visual stream, and the onset of the audio stream of the male voice occurred *following* the onset of the male visual stream. During the Adaptation phase, the temporal distance between the onset of audio and visual components was always ±300 ms. Between subsequent presentations there was a pause of 1300–1700 ms, determined on a presentation-by-presentation basis. During the adaptation period, participants were instructed to simply pay attention to the temporal relationship between audio and visual presentations, an instruction similar to that typically used (Heron et al., 2010, 2012; Roseboom and Arnold, 2011).

Subsequent to the Adaptation period, participants completed the Test phase in which they were required to make synchrony/asynchrony judgments regarding presentations of the audio-visual stimuli which they had viewed during the Adaptation phase. In the Test phase the temporal relationship between audio and visual components was manipulated across nine levels (−433, −333, −233, −133, 0, 133, 233, 333, 433 ms; negative numbers indicating audio occurred before vision). Prior to each Test trial presentation, participants viewed an adaptation top-up sequence in which two presentations of each of the previously viewed adapting configurations from the Adaptation phase were again presented. Following this four presentation sequence, participants were informed that they would be required to respond to the next presentation by a change in the central fixation cross from red to green (see Figure 1; Movie S1 in Supplementary Material).

As there were two audio-visual stimuli, and two possible audio-visual temporal relationships (audio leading vision; audio trailing vision), there were four possible stimulus configurations. Each experimental session concurrently adapted the two different audio-visual stimulus combinations to opposite temporal relationships, creating two experimental conditions (male audio leads vision with female audio lags vision; and male audio lags vision with female audio leads vision). For each condition, participants completed four blocks of 72 trials; 36 Test trials for each of the two audio-visual stimulus combinations, with four repeats at each of the nine audio-visual temporal offsets. The order of completion of trials in a given block was pseudo-random. Each condition required the completion of 288 trials, 576 trials across all four conditions. Each of the eight blocks of trials took ∼25 min to complete. Participants completed the different conditions over a 2 day period with the four blocks of a given condition completed in a single day.

### Results

Participants’ PSS’s were estimated separately for each of the stimulus identities, for each of the two possible adaptation timing relationships. The PSS was taken as the peak of a truncated Gaussian function fitted to participants’ response distributions (as done in Roseboom and Arnold, 2011) obtained from synchrony/asynchrony judgments completed during Test phases (see Supplemental Material for PSS’s estimated as the average of upper and lower boundaries of a distribution fitted by the difference of two cumulative Gaussian functions based on methods demonstrated in Yarrow et al., 2011b). We also took the SD of the fitted functions as a measure of the width of the response distribution. This value is often used as an indicator of the precision with which participants are responding.

**Figure 2:**
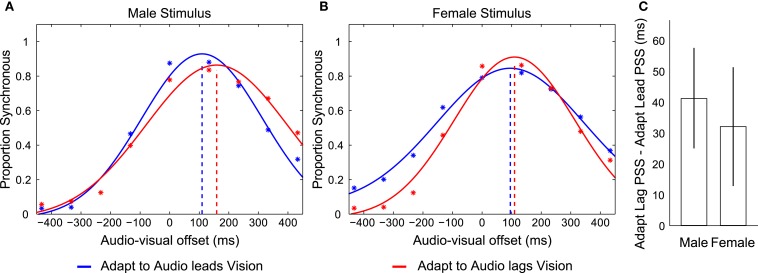
**Depictions of data from Experiment 1**. **(A,B)** Distributions of reported audio and visual synchrony for eight participants in trials following adaptation to an audio leading vision relationship (blue) and an audio lagging vision relationship (red) for the Male and Female stimulus types. Broken vertical lines indicate the point of subjective synchrony (PSS). If the red vertical line is placed to the right of the blue line (i.e., more positive) it indicates that an appropriate direction of adaptation was achieved. Adapt to Audio leads Vision refers to trials in which the exposed timing relationship between audition and vision during the Adaptation phase for the given stimulus was such that audition was presented prior to vision by 300 ms. Adapt to Audio lags Vision refers to the reverse case, where the exposed timing relationship during adaptation was such that audition was presented following vision by 300 ms. Note that participants concurrently adapted to opposite audio-visual timing relationships for each stimulus during a given set of trials such that they adapted to audio leads vision for the Male stimulus while concurrently adapting to audio lags vision for the Female stimulus, or vice versa. **(C)** Difference in PSS between adapting to an audio leading vision compared to audio lagging vision relationship for each stimulus, for each participant, averaged across the eight participants. Error bars indicate ±1 SEM.

We conducted a repeated measures analysis of variance (ANOVA) using the individual PSS’s from each of the four possible audio-visual-adaptation relationships (Male and Female, adapting to audio leading and lagging vision relationship; the average of these values for eight participants are shown in Figure 2). This analysis revealed a main effect of the adapted timing relationship (*F*_1,7_ = 9.705, *p* = 0.017) such that participants’ PSS’s were significantly larger in trials following adaptation to audio lagging vision (Lag = 136.653; SEM = 17.408) compared with trials following adaptation to audio leading vision (Lead = 100.114; SEM = 18.856). There was also a main effect of identity (*F*_1,7_ = 9.228, *p* = 0.019) such that the PSS’s for the male stimulus (Male = 138.987; SEM = 14.814) were larger than for the female stimulus (Female = 97.781; SEM = 20.665), but there was no interaction between stimulus identity and adapting relationship (*F*_1,7_ = 0.115, *p* = 0.745). We also conducted a repeated measures ANOVA on the SD data of the fitted functions. This revealed a significant main effect of the different stimuli (*F*_1,7_ = 9.78, *p* = 0.017) such that the SD was larger for responses regarding the Female stimulus (mean = 248.694; SEM = 21.914) than the Male (mean = 211.969; SEM = 22.734). However, there was no difference in SD’s between adaptation conditions, nor any interaction between adaptation condition and stimulus type (*F*’s < 0.722; *p*’s > 0.424). Overall, these results are consistent with participants having concurrently adapted to opposite temporal relationships for the different stimulus identities regardless of spatial overlap of presentation.

## Experiment 2

The results of Experiment 1 are consistent with those previously reported by Roseboom and Arnold (2011); specifically, that multiple concurrent temporal recalibrations of audio-visual speech can be constrained by the content of the stimulus, male or female identity of the speaker. This result is found whether the stimuli are presented from the same spatial location during both the Adaptation and Test phases (Experiment 1) or not (Roseboom and Arnold, 2011). Critically, the only difference between those two results is that in Experiment 1 of this study, there is no difference in the presentation location at any stage during the experiment. In the previous study by Roseboom and Arnold (2011), the specificity of temporal recalibrations by identity was established by testing the different identity stimuli at different spatial locations from that in which they were presented during the adaptation period. Consequently, the results of Experiment 1 confirm the conclusions of Roseboom and Arnold (2011).

**Figure 3:**
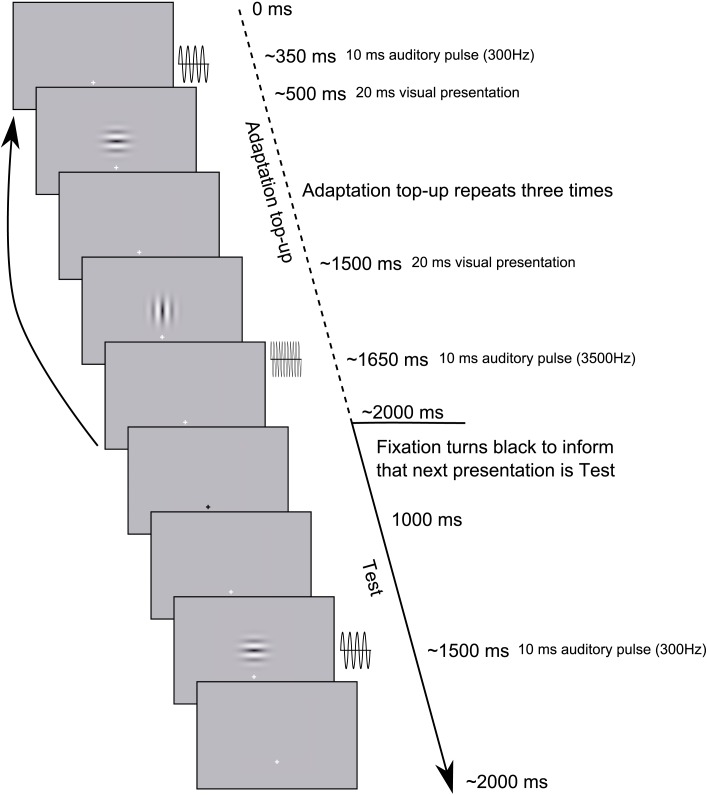
**Example Test trial sequence from Experiment 2**. Each trial began with an Adaptation top-up period during which three repeats of each of the adapting relationships from the previously presented Adaptation phase were repeated. Following the top-up period, participants were informed by a transient change in the fixation cross from white to black that the next presentation would be a Test presentation to which they would have to respond. The Adaptation phase consisted of 30 repeats of each stimulus configuration, as depicted in the Adaptation top-up period, before proceeding onto the Adaptation top-up/Test presentation cycle.

However, one possible criticism of the results presented in Experiment 1 is that, while the *overall* position of presentation did not differ between the different stimulus presentations, the spatial properties of the different faces were not precisely matched. Indeed, by using video clips obtained from real individuals with clearly male and female identities such differences are bound to be introduced as the face dimensions of different genders are not identical (Burton et al., 1993). Therefore, it may be that while overall presentation location did not vary between the stimuli, small scale differences in spatial configuration may have provided enough information to cue differential temporal recalibration. This speculation, combined with the previous failure to obtain results supporting multiple concurrent recalibrations using more basic stimuli (Heron et al., 2012), makes it unclear whether the constraint by content is unique to complex stimuli containing many small scale differences in spatial configuration, or whether it is possible for truly spatially overlapping stimuli. To investigate this issue we set up an experiment similar to that of Heron et al. (2012) using simple stimuli. The visual stimuli were defined by either vertically or horizontally oriented Gabors and the auditory stimuli were high or low pitch tones (see Figure 3; Movie S2 in Supplementary Material for example). There was no difference in spatial location of presentation for the different visual or auditory stimuli during any phase of the experiment. As such, this experiment was designed to explicitly investigate whether multiple, concurrent, audio-visual temporal recalibrations are possible for simple stimuli constrained only by featural differences.

### Methods

The apparatus was similar to that used in Experiment 1, though the refresh rate of the monitor was 100 Hz. Five participants, naïve as to experimental purpose, completed the experiment. All reported normal or corrected to normal vision and hearing. Written informed consent was obtained from all participants.

The visual stimuli consisted of a vertically or horizontally oriented Gabor patch (SD = 0.7°, background luminance 62 cd/m^2^, carrier spatial frequency of 3.5 cycles/degree, Michelson contrast ∼1) centered 2.4° of visual angle above a white (123 cd/m^2^) central fixation point (0.4° of visual angle in width and height; see Figure 3, for depiction). Individual visual stimulus presentations were 20 ms in duration. Auditory signals consisted of a 10 ms pulse, containing 2 ms cosine onset and offset ramps of 300 or 3500 Hz sine-wave carrier at ∼55 db SPL. As such, there were four possible audio-visual stimulus pairs; vertical Gabor and 300 Hz sound, vertical Gabor with 3500 Hz sound, horizontal Gabor with 300 Hz sound, and horizontal Gabor with 3500 Hz sound.

### Procedures

As in Experiment 1, the experiment consisted of two phases, Adaptation and post-adaptation Test. During the Adaptation phase participants observed 30 presentations of each of two audio-visual combinations, sequentially alternating between the two (see Movie S2 in Supplementary Material for example trial sequence). The two audio-visual combinations possessed opposite audio-visual temporal relationships, such that, for example (as in Figure 3; Movie S2 in Supplementary Material), a low pitch sound occurred *prior* to a horizontal Gabor, and a high pitch sound occurred *following* a vertical Gabor. During the Adaptation phase, the temporal distance between the onset of audio and visual components was always ±150 ms. Between subsequent presentations there was a pause of 1000–2000 ms, determined on a presentation-by-presentation basis.

Prior to commencing the experiment, participants were shown what the different audio and visual stimuli looked and sounded like. They were then informed explicitly that they would be watching the presentation of two distinct audio-visual pairs and told, for example, that one pair may consist of the vertical visual stimulus and the high pitch audio stimulus, while the other would consist of the horizontal visual stimulus and the low pitch audio stimulus. Moreover, they were informed that the different pairs would possess different audio-visual temporal relationships such that for one pair the visual stimulus would appear prior to the audio stimulus, while for the other pair the visual stimulus would appear following the audio stimulus. They were instructed that their task during the Adaptation period was to pay attention to the temporal discrepancies between audio and visual components for each of the different pairs, a variation on instructions that have previously been shown to be successful in inducing audio-visual temporal recalibration for single audio-visual pairs (Heron et al., 2010). See also Supplemental Experiment 1 for results of a task using slightly different instructions.

Subsequent to the Adaptation period, participants completed the Test phase in which they were required to make synchrony/asynchrony judgments regarding presentations of the audio-visual stimuli which they had viewed during the Adaptation phase. In the Test phase, audio-visual stimuli were always presented in the same pitch-orientation combinations as had been viewed during the immediately previous Adaptation phase, and the temporal relationship between audio and visual components was manipulated across 11 levels (50 ms steps from −250 to +250). Prior to each Test trial presentation, participants viewed an adaptation top-up sequence in which three presentations of each of the previously viewed adapting configurations from the Adaptation phase were again presented. Following this six presentation sequence, participants were informed that they would be required to respond to the next presentation by a change in the central fixation cross from white to black for 1000 ms (see Movie S2 in Supplementary Materials for example trial sequence).

As there were four audio-visual stimulus combinations, and two possible audio-visual temporal relationships (audio leading vision; audio trailing vision), there were eight possible stimulus configurations. Each experimental session concurrently adapted two different audio-visual stimulus combinations to opposite temporal relationships, creating four experimental conditions (low pitch-horizontal audio leads vision and high pitch-vertical audio lags vision; low pitch-horizontal audio lags vision and high pitch-vertical audio leads vision; high pitch-horizontal audio leads vision and low pitch-vertical audio lags vision; and high pitch-horizontal audio lags vision and low pitch-vertical audio leads vision). For each condition, participants completed four blocks of 88 trials; 44 Test trials for each of the two audio-visual stimulus combinations, with four repeats at each of the 11 audio-visual temporal offsets. The order of completion of trials in a given block was pseudo-random. Each condition required the completion of 352 trials, 1408 trials across all four conditions. Each of the 16 blocks of trials took ∼20 min to complete. Participants completed the different conditions in a pseudo-random order over a 4 day period with the four blocks of a given condition completed in a single day.

### Results

Participants’ PSS’s were estimated separately for each of the four audio-visual combinations, at each of the two possible adaptation timing relationships. The PSS was taken as the peak of a truncated Gaussian function fitted to participants’ response distributions (as done in Roseboom and Arnold, 2011) obtained from audio-visual synchrony/asynchrony judgments for that condition completed during Test phases (see Supplemental Material for PSS’s estimated as the average of upper and lower boundaries of a distribution fitted by the difference of two cumulative Gaussian functions based on methods demonstrated in Yarrow et al., 2011b). Again, we also took the standard deviation of the fitted function as a measure of the precision with which participants are responding.

**Figure 4:**
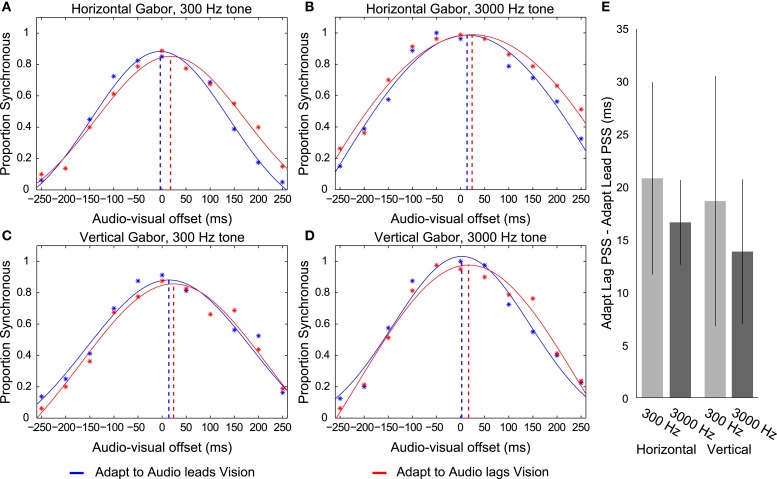
**Depictions of data from Experiment 2**. **(A–D)** Distributions of reported audio and visual synchrony for five participants in trials following adaptation to an audio leading vision relationship (blue) and an audio lagging vision relationship (red) for the four different stimulus combinations. Broken vertical lines indicate the point of subjective synchrony (PSS). If the red vertical line is placed to the right of the blue line (i.e., more positive) it indicates that an appropriate direction of adaptation was achieved. Adapt to Audio leads Vision refers to trials in which the exposed timing relationship between audition and vision during the Adaptation phase for the given stimulus was such that audition was presented prior to vision by 150 ms. Adapt to Audio lags Vision refers to the reverse case, where the exposed timing relationship during adaptation was such that audition was presented following vision by 150 ms. Note that participants concurrently adapted to opposite audio-visual timing relationships for each stimulus during a given set of trials such that, for example, they adapted to audio leads vision for the Horizontal Gabor and 300 Hz tone combination while concurrently adapting to audio lags vision for the Vertical Gabor and 3000 Hz combination. **(E)** Difference in PSS between adapting to an audio leading vision compared to audio lagging vision relationship for each stimulus, for each participant, averaged across the eight participants. Error bars indicate ±1 SEM.

We conducted a repeated measures ANOVA using the individual PSS’s from each of the eight possible audio-visual-adaptation relationships (see Figure 4 for overall data). This analysis revealed a main effect of the adapted timing relationship (*F*_1,4_ = 25.069, *p* = 0.007), such that participants’ PSS’s were significantly larger in trials following adaptation to audio lagging vision (mean = 28.343; SEM = 18.099) compared with trials following adaptation to audio leading vision (mean = 10.883; SEM = 15.915). There was no main effect of different visual stimulus type (*F*_1,4_ = 0.262, *p* = 0.636) but perhaps a trending influence of different auditory stimulus type (*F*_1,4_ = 5.33, *p* = 0.082). However, there was no significant interaction between stimulus types and adaptation timing relationship (*F*’s < 3.364; *p*’s > 0.141). We also conducted a repeated measures ANOVA on the SD data of the fitted functions. This revealed no significant difference between different stimuli or adaptation conditions (*F*’s < 5.135; *p*’s > 0.086; overall mean SD = 143.98 ms). Overall, these results are consistent with participants having concurrently adapted to opposite temporal relationships for the different stimulus combinations regardless of spatial overlap of presentation.

## General Discussion

The purpose of this study was to determine whether it is possible to obtain multiple concurrent audio-visual temporal recalibrations when stimuli differ in featural content, but not in overall spatial location of presentation at any point during the experimental procedure. This was done in an attempt to resolve the difference in results obtained by two recent studies; Roseboom and Arnold (2011) demonstrated that multiple audio-visual temporal recalibrations could be constrained by featural information of the stimuli, while Heron et al. (2012) suggested that different recalibrations could only be constrained by spatial information. Here, we revealed that two concurrent and opposite audio-visual temporal recalibrations are possible regardless of spatial overlap for both naturally compelling (Experiment 1) and arbitrary stimulus combinations (Experiment 2).

### Inconsistencies with Heron et al. (2012)

Experiment 1 of this study explicitly addressed one of the primary differences between the two previous studies (Roseboom and Arnold, 2011 and Heron et al., 2012) – whether a difference in spatial location during the adaptation phase of the experiment is required. However, Experiment 2 might be considered more of a conceptual replication of the experiment from Heron et al. (2012) investigating a case of pure content/featural difference. In that experiment, Heron et al. (2012) found no evidence for multiple concurrent recalibrations, while the results of Experiment 2 of this study clearly demonstrate such an effect. This inconsistency may be attributable to minor differences in experimental paradigm between the two studies. These differences are largely superficial, but here we will speculate that they may have contributed to the overall difference.

#### Basic stimulus properties

First, the visual stimuli we used in Experiment 2 were defined by orientation rather than spatial frequency (as in Heron et al., 2012). Further, the audio stimuli were defined by 300 and 3000 Hz sine carrier pure tones, rather than 500 and 2000 Hz. These differences, while minor, may have facilitated participant’s segmentation of the adapting stream into clear audio-visual pairs (e.g., vertical orientated visual paired with 300 Hz tone) to be recalibrated, while the differences in spatial frequency used by Heron et al. (2012) may not have been as clear.

#### Temporal structure of adaptation presentations

Along the same lines, the temporal structure of presentation was slightly different in our experiment compared with that of Heron et al. (2012) In their study, during the adaptation phase, successive audio-visual pairs were separated by an interval of between 500 and 1000 ms. In our study, this value was between 1000 and 2000 ms. Given that effective binding of audio and visual events becomes impossible at repetition rates of greater than 2–3 Hz (Fujisaki and Nishida, 2010), the inter presentation interval used by Heron et al. (2012) may have been brief enough to have sometimes caused confusion as to which audio and visual events comprised a specific pair. A related fact that may support this kind of conclusion is that when using audio-visual speech, such as in Experiment 1 of this study and in Roseboom and Arnold (2011), the repetition rate is much lower as speech stimuli are much longer (in this study a maximum of 800 ms) than the simple stimuli (in this study a maximum of 160 ms). This temporal factor, rather than any special ecological validity of audio-visual speech (Yuan et al., 2012), may in fact account for the apparent comparative ease with which concurrent and opposite temporal recalibrations can be obtained for speech relative to simple stimuli. We believe this speculation deserves further investigation.

#### Experimental instructions

Finally, the experimental instructions used in Experiment 2 of this study differed slightly from those reportedly used by Heron et al. (2012). In Experiment 2 of our study we provided participants with extensive information about the task and explicitly informed them of which audio and visual signals comprised a pair during a given experimental condition. In the study by Heron et al. (2012) participants were told only to attend to the temporal relationship between audio and visual stimuli. Indeed when we employed instructions similar to those used by Heron et al. (2012) using five naïve participants, we found no reliable adaptation effects (see Supplemental Experiment 1). Consequently, it seems likely that this factor also contributed to determining the appropriate audio-visual pair to recalibrate to a given audio-visual temporal relationship (note, however, that respectively four and three of the six participants used in experiment one and two by Heron and colleagues were the authors).

### The comparative role of space and features

An important point to make is the fact that content information can constrain multiple temporal recalibrations in the absence of spatial disparity is not to say that spatial relation has no role in multiple concurrent recalibrations or in temporal recalibration generally. Indeed previous evidence strongly supports the role of spatial disparity in constraining temporally recalibrated estimates of synchrony when the task and stimulus configurations provide a clear reason to do so (Yarrow et al., 2011a; Heron et al., 2012; Yuan et al., 2012). However, when there is no requirement to be specific about spatial relationship, as when there is only a single possible audio-visual relationship presented and the task demands require you to treat it as such (Keetels and Vroomen, 2007), when there is another strongly compelling cue as to the appropriate audio-visual relationship (e.g., identity; Roseboom and Arnold, 2011), or when there is no useful spatial information available (such as in this study), spatial cues are not required to determine the appropriate audio and visual signal combination to recalibrate. Certainly, if one were to equate the strength of some set of spatial, content, and task demand cues such that they were equally contributing to determination of the specific audio-visual relationship then it would be possible to examine a direct trade-off between these different factors. The most appropriate task to use in order to accomplish this is not entirely clear as there would be many possible dimensions of interaction, however we believe it to be conceptually possible. The results of a recent study (Yuan et al., 2012) support this premise. Although in that study the strength of different cues was not directly equated, they did compare the magnitude of context and spatially constrained recalibrations when the spatial location of auditory presentations was clear (presented from spatially co-localized loud speakers) with that when auditory presentations were from spatially non-localized headphones. These comparisons revealed that the relative magnitude of temporal recalibration effects, as defined by spatial or context based cues, was modulated by whether the spatial information from auditory cues was strong (loud speaker condition) or less informative (headphone condition).

For achieving useful outcomes in real world scenarios it is likely that the strength of a given cue is determined by interplay between many factors including top-down influences from attention (Heron et al., 2007), along with stimulus properties that are typically associated with cue combination (signal reliability; e.g., Hillis et al., 2002; Battaglia et al., 2003; and covariance; e.g., Parise et al., 2012) and prior knowledge of the likelihood those signals are related (Guski and Troje, 2003; Miyazaki et al., 2006; Vatakis and Spence, 2007, 2008; see Ma, 2012 for a recent review of possible statistical implementations in these kinds of scenarios).

### What does this mean for putative mechanisms of temporal recalibration?

It may be important to differentiate how different audio-visual components are selected as appropriate pairs to be recalibrated from how a given temporal recalibration may be implemented. With regards to this latter point, several proposals have been made (e.g., selective modulation of unisensory processing speed, Di Luca et al., 2009; Navarra et al., 2009; modulation of prior likelihood distributions, Yamamoto et al., 2012; asymmetrical change in synchrony judgment criteria, Yarrow et al., 2011b; adaptation of delay sensitive neurons, Roach et al., 2011; Note that these possibilities are not necessarily mutually exclusive). That the recalibration effect can be constrained by what would typically be considered highly complex information, such as identity of a speaker, creates problems in resolving the effect we report here with some of these proposals. Generally speaking, the results of this study support a characterization of audio-visual temporal recalibration as being primarily a decision-level effect that occurs as a result of a selective change in synchrony criteria on the side of the exposed asynchrony (Yarrow et al., 2011b) for a specific audio-visual stimulus. An alternative possibility is that the multiple concurrent recalibration effect is representative of a process that only acts to constrain the operation of a more basic and direct mechanism of temporal recalibration. Making this kind of distinction suggests a two stage account of multiple temporal recalibration and may allow design of paradigms wherein the putative operations are in conflict (e.g., Yamamoto et al., 2012). These possibilities remain firmly speculative at this point and further clarification is required before any firm conclusions can be drawn.

Another potentially interesting direction of investigation regards the number of possible concurrent recalibrations that can be maintained. In this and previous studies addressing multiple concurrent recalibrations (Roseboom and Arnold, 2011; Heron et al., 2012) only two different audio-visual temporal relationships were used; one with audio leading vision and the other with audio lagging vision. Such an arrangement is preferable under highly constrained experimental conditions as it will maximize possible differences between the two experimental conditions. However, whether more than two temporal recalibrations can be maintained is an interesting question that may shed light on the nature of the broader mechanism. It has previously been established that the PSS for different audio-visual event pairs can differ by the type of signals used (e.g., speech compared with music; Vatakis and Spence, 2006) and the conditions under which they are judged (e.g., temporally sparse compared with more temporally cluttered; Roseboom et al., 2009; see van Eijk et al., 2008 for a review of studies examining subjective synchrony with different stimuli and under different conditions). In this study we adapted the temporal relationship for specific audio-visual pairs over a brief exposure period. Whether the process underlying the observed change in subjective synchrony is associated with longer term determinants of synchrony, or is only a short term adaptive process, is not entirely clear. However, it has recently been demonstrated that, rather than simply dissipating over time, a recalibrated sense of synchrony is maintained until sufficient exposure to contradictory evidence (Machulla et al., 2012). This result may be consistent with the idea that short term asynchrony exposure is simply the action of general processes for determining the relationship between specific audio and visual signals.

## Conclusion

Determining the appropriate way to interpret an incoming stream of multisensory events is a critical and difficult task for the human perceptual system. In complex sensory environments it makes sense to be flexible and adaptive. Here we add to previous demonstrations showing that humans can not only adjust to inter-sensory temporal discrepancies (Fujisaki et al., 2004; Vroomen et al., 2004), but can do so selectively (Roseboom and Arnold, 2011; Heron et al., 2012). This selectivity can be constrained by many factors including apparent spatial (Heron et al., 2012) and featural (Roseboom and Arnold, 2011) correspondence. In a complex environment with many cues as to the correspondence between different sensory signals, being able to use important featural information, such as the identity of a speaker, is an attractive strategy. Here we have demonstrated that it is possible to use such rich sources of information in the absence of any spatial discrepancy for both naturally compelling and arbitrary stimulus combinations. How such information is utilized in creating an altered sense of timing remains an unresolved question, but these results suggest that audio-visual temporal recalibration is the result of complex decisional processes taking into account many aspects of sensory events including spatial and featural correspondence along with prior knowledge of likely relatedness.

## Conflict of Interest Statement

The authors declare that the research was conducted in the absence of any commercial or financial relationships that could be construed as a potential conflict of interest.

## Supplementary Material

The Supplementary Material for this article can be found online at http://www.frontiersin.org/Perception_Science/10.3389/fpsyg.2013.00189/abstract

Supplementary Movies S1 and S2 **Please note that the supplementary movies provided are not the actual stimuli used in the experiments**. The movies are only approximations intended to give the reader an impression of the trial presentation appearance. Due to technical constraints we cannot guarantee that these movies precisely match the spatial and temporal properties described for the actual experimental stimuli in the Section “Materials and Methods.”

## Appendix

### Supplemental results

#### Supplemental experiment 1

##### Methods

The methods of Supplemental Experiment 1 were identical to that of Experiment 2 with the following exceptions. Participants consisted of five new participants, all of whom were naïve as to experimental purpose. Unlike in Experiment 2, participants were given no explicit information about the presentation sequence during the Adaptation phase, they were simply informed to pay attention to the temporal relationship between audio and visual presentations. These instructions approximate those reported to have been used by Heron et al. (2012).

##### Results

Results were analyzed as in Experiment 2, with participants’ PSS’s estimated separately for each of the four audio-visual combinations, at each of the two possible adaptation timing relationships. The PSS was taken as the peak of a truncated Gaussian function fitted to participants response distributions obtained from audio-visual synchrony/asynchrony judgments for that condition completed during Test phases (see below for results when PSS’s were estimated as the average of upper and lower boundaries of a distribution fitted by the difference of two cumulative Gaussian functions based on methods demonstrated in Yarrow et al., 2011b).

We conducted a repeated measures ANOVA using the individual PSS’s from each of the eight possible audio-visual-adaptation relationships. This analysis revealed no effect of the adapted timing relationship (*F_1,4_* = 0.01, *p* = 0.921), nor any effects of different visual or auditory stimulus type (*F*’s < 4.82; *p*’s > 0.093). We also conducted a repeated measures ANOVA using the SD of the functions fitted to response distributions. There was no effect of adaptation or stimulus conditions on the width of the fitted functions (*F*’s < 3.582; *p*’s > 0.131). These results suggest that the instructions provided to participants may be critical to obtaining different concurrent temporal recalibrations. This outcome is broadly consistent with previous findings indicating that where participants direct their attention during the adaptation phase of the experiment can have a significant influence on the magnitude of the recalibration effect (Heron et al., 2010; Tanaka et al., 2011). In Experiment 2, we explicitly instructed participants to attend specifically to the different AV combinations and their respective audio-visual asynchronies. However, in Supplemental Experiment 1, participants were given no instructions regarding any difference of the stimuli. The use of arbitrary stimulus combinations was, of itself, unlikely to promote perception of the different combinations as distinct from one another. By contrast, similar experimental instructions to those used in Supplemental Experiment 1 were also given in Experiment 1. In that case the stimuli were two different clips of real audio-visual speech. Such stimuli may implicitly contain the appropriate information to encourage participants to consider each audio-visual stimulus as distinct from the other. However, the different temporal properties of the stimuli may also be a factor (see General Discussion in the main text).

##### Fitting response distributions as the difference of two cumulative Gaussian functions

When using synchrony/asynchrony (simultaneity) judgments such as we have used in this study, it is often considered standard practice to fit the obtained response distributions with a probability density function, such as the Gaussian function. However, recently (Yarrow et al., 2011b) it was proposed that an alternative method, fitting the response distribution with two cumulative Gaussian functions, may be superior^1^. The reasons for this conclusion remain a matter of debate and are certainly outside the scope of the present study. However, in this study we provide results obtained under both approaches for the purpose of comparison for those inclined to do so.

#### Experiment 1

Participants’ PSS’s were estimated separately for each of the stimulus identities, for each of the two possible adaptation timing relationships. The PSS was taken as the average of upper and lower boundaries of a distribution fitted by two cumulative Gaussian functions (Yarrow et al., 2011b; Yuan et al., 2012) obtained from synchrony/asynchrony judgments completed during Test phases.

We conducted a repeated measures ANOVA using the individual PSS’s from each of the four possible audio-visual-adaptation relationships (Male and Female, adapting to audio leading and lagging vision relationship). This analysis revealed a main effect of the adapted timing relationship (*F_1,7_* = 6.262, *p* = 0.041), such that participants’ PSS’s were significantly larger in trials following adaptation to audio lagging vision (Lag = 140.34; SEM = 20.662) compared with trials following adaptation to audio leading vision (Lead = 108.27; SEM = 22.468). There was no main effect of stimulus identity (*F_1,7_* = 0.140, *p* = 0.719) nor interaction between identity and adaptation timing (*F_1,7_* = 2.26, *p* = 0.176). We conducted a repeated measures ANOVA using the SD’s of the functions fitted to the upper and lower bounds of response distributions. There was no effect of adaptation, stimulus conditions, or boundary side (audio leads or lags vision) on the width of the fitted functions (*F*’s < 3.878; *p*’s > 0.090). Overall, these results are consistent with those reported in the main text indicating that participants concurrently adapted to opposite temporal relationships for the different stimulus identities regardless of spatial overlap.

#### Experiment 2

The Supplemental Results of Experiment 2 were analyzed in a similar way to that shown in the Supplemental Results of Experiment 1. We again conducted a repeated measures ANOVA using the individual PSS’s from each of the eight possible audio-visual-adaptation relationships. This analysis revealed a main effect of the adapted timing relationship (*F_1,4_* = 12.775, *p* = 0.023), such that participants’ PSS’s were significantly larger in trials following adaptation to audio lagging vision (mean = 28.915; SEM = 18.488) compared with trials following adaptation to audio leading vision (mean = 7.257; SEM = 13.420). There was no main effect of visual or auditory stimulus type (*F*’s < 1.254; *p*’s > 0.326). We again conducted a repeated measures ANOVA using the SD’s of the functions fitted to the upper and lower bounds of response distributions. There was no effect of adaptation, stimulus conditions, or boundary side on the width of the fitted functions (*F*’s < 4.540; *p*’s > 0.077). Overall, these results are consistent with those from reported in the main text supporting the premise that participants concurrently adapted to opposite temporal relationships for different stimulus combinations regardless of spatial overlap.

#### Supplemental experiment 1

The Supplemental results of Supplemental Experiment 1 were analyzed in the same fashion as those for Experiment 2. As for the results reported above for Supplemental Experiment 1, conducting a repeated measures ANOVA using the individual PSS’s from each of the eight possible audio-visual-adaptation relationships reveals no effect of the adapted timing relationship (*F_1,4_* = 1.482, *p* = 0.290), nor any effects of different visual or auditory stimulus type (*F*’s < 1.42; *p*’s > 0.29). We again conducted a repeated measures ANOVA using the SD’s of the functions fitted to the upper and lower bounds of response distributions. There was no effect of adaptation, stimulus conditions, or boundary side on the width of the fitted functions (*F*’s < 2.501; *p*’s > 0.189).

^1^See Yarrow et al. (2011b) for a detailed description of this approach and a comparison with the standard practice. In short, this approach can be summarized as fitting two cumulative probability functions (in this case cumulative Gaussians) that each describe one of the two sides of the distribution. These functions provide estimates of the decision boundaries for temporal order between the audio and visual signals (i.e., decision regarding audio leading vision on one side and audio lagging vision on the other).

## References

[B1] Alais D. Burr D. (2004). The ventriloquist effect results from near-optimal bimodal integration. Curr. Biol. 14, 257–262 10.1016/S0960-9822(04)00043-0 14761661

[B2] Arnold D. H. Tear M. Schindel R. Roseboom W. (2010). Audio-visual speech cue combination. PLoS ONE 5:e10217 10.1371/journal.pone.0010217 20419130 PMC2855706

[B3] Arnold D. H. Yarrow K. (2011). Temporal recalibration of vision. Proc. R. Soc. Lond. B Biol. Sci. 278, 535–538 10.1098/rspb.2010.1396 PMC3025680 20826481

[B4] Ayhan I. Bruno A. Nishida S. Johnston A. (2009). The spatial tuning of adaptation-based time compression. J. Vis. 9, 1–12 10.1167/9.13.1 20053065

[B5] Battaglia P. W. Jacobs R. A. Aslin R. N. (2003). Bayesian integration of visual and auditory signals for spatial localization. J. Opt. Soc. Am. 20, 1391–1397 10.1364/JOSAA.20.001391 12868643

[B6] Bennett R. G. Westheimer G. (1985). A shift in the perceived simultaneity of adjacent visual stimuli following adaptation to stroboscopic motion along the same axis. Vision Res. 25, 565–569 10.1016/0042-6989(85)90161-0 4060609

[B7] Bruno A. Ayhan I. Johnston A. (2010). Retinotopic adaptation-based visual duration compression. J. Vis. 10, 1–18 10.1167/10.7.1413 20884495

[B8] Burr D. Corsale B. (2001). Dependency of reaction times to motion onset on luminance and chromatic contrast. Vision Res. 41, 1039–1048 10.1016/S0042-6989(01)00072-4 11301077

[B9] Burton A. M. Bruce V. Dench N. (1993). What’s the difference between men and women? Evidence from facial measurement. Perception 22, 153–176 10.1068/p220153 8474841

[B10] Di Luca M. Machulla T. K. Ernst M. O. (2009). Recalibration of multisensory simultaneity: cross-modal transfer coincides with a change in perceptual latency. J. Vis. 9, 1–16 10.1167/9.13.1 20053098

[B11] Ernst M. Banks M. (2002). Humans integrate visual and haptic information in a statistically optimal fashion. Nature 415, 429–433 10.1038/415429a 11807554

[B12] Evans K. K. Treisman A. (2010). Natural cross-modal mappings between visual and auditory features. J. Vis. 10, 1–12 10.1167/10.3.10 20143899 PMC2920420

[B13] Fujisaki W. Nishida S. (2010). A common perceptual temporal limit of binding synchronous inputs across different sensory attributes and modalities. Proc. R. Soc. Lond. B Biol. Sci. 277, 2281–2290 10.1098/rspb.2010.0243 PMC2894908 20335212

[B14] Fujisaki W. Shimojo S. Kashino M. Nishida S. (2004). Recalibration of audiovisual simultaneity. Nat. Neurosci. 7, 773–778 10.1038/nn1268 15195098

[B15] Guski R. Troje N. F. (2003). Audiovisual phenomenal causality. Percept. Psychophys. 65, 789–800 10.3758/BF03194815 12956586

[B16] Hanson J. V. Heron J. Whitaker D. (2008). Recalibration of perceived time across sensory modalities. Exp. Brain Res. 185, 347–352 10.1007/s00221-008-1282-3 18236035

[B17] Harrar V. Harris L. R. (2008). The effect of exposure to asynchronous audio, visual, and tactile stimulus combinations on the perception of simultaneity. Exp. Brain Res. 186, 517–524 10.1007/s00221-007-1253-0 18183377

[B18] Heron J. Roach N. W. Hanson J. V. McGraw P. V. Whitaker D. (2012). Audiovisual time perception is spatially specific. Exp. Brain Res. 218, 477–485 10.1007/s00221-012-3038-3 22367399 PMC3324684

[B19] Heron J. Roach N. W. Whitaker D. Hanson J. V. M. (2010). Attention regulates the plasticity of multisensory timing. Eur. J. Neurosci. 31, 1755–1762 10.1111/j.1460-9568.2010.07194.x 20584179 PMC3362737

[B20] Heron J. Whitaker D. McGraw P. V. Horoshenkov K. V. (2007). Adaptation minimizes distance-related audiovisual delays. J. Vis. 7, 1–8 10.1167/7.6.1 17997633

[B21] Hillis J. M. Ernst M. O. Banks M. S. Landy M. S. (2002). Combining sensory information: mandatory fusion within, but not between, senses. Science 298, 1627–1630 10.1126/science.1075396 12446912

[B22] Johnston A. Arnold D. H. Nishida S. (2006). Spatially localised distortions of perceived duration. Curr. Biol. 16, 472–479 10.1016/j.cub.2006.01.032 16527741

[B23] Keetels M. Vroomen J. (2007). No effect of auditory-visual spatial disparity on temporal recalibration. Exp. Brain Res. 182, 559–565 10.1007/s00221-007-1012-2 17598092 PMC2190788

[B24] King A. J. (2005). Multisensory integration: strategies for synchronization. Curr. Biol. 15, R339–341 10.1016/j.cub.2005.04.022 15886092

[B25] Kopinska A. Harris L. R. (2004). Simultaneity constancy. Perception 33, 1049–1060 10.1068/p5169 15560507

[B26] Lennie P. (1981). The physiological basis of variations in visual latency. Vision Res. 21, 815–824 10.1016/0042-6989(81)90180-2 7314459

[B27] Ma W. J. (2012). Organizing probabilistic models of perception. Trends Cogn. Sci. (Regul. Ed.) 16, 511–518 10.1016/j.tics.2012.08.010 22981359

[B28] Machulla T.-K. Di Luca M. Frölich E. Ernst M. O. (2012). Multisensory simultaneity recalibration: Storage of the aftereffect in the absence of counterevidence. Exp. Brain Res. 217, 89–97 10.1007/s00221-011-2976-5 22207361

[B29] Miyazaki M. Yamamoto S. Uchida S. Kitazawa S. (2006). Bayesian calibration of simultaneity in tactile temporal order judgment. Nat. Neurosci. 9, 875–877 10.1038/nn1712 16732276

[B30] Navarra J. García-Morera J. Spence C. (2012). Temporal adaptation to audiovisual asynchrony generalizes across different sound frequencies. Front. Psychol. 3:152 10.3389/fpsyg.2012.00152 22615705 PMC3351678

[B31] Navarra J. Hartcher-O’Brien J. Piazza E. Spence C. (2009). Adaptation to audiovisual asynchrony modulates the speeded detection of sound. Proc. Natl. Acad. Sci. U.S.A. 106, 9169–9173 10.1073/pnas.0810486106 19458252 PMC2695059

[B32] Navarra J. Soto-Faraco S. Spence C. (2007). Adaptation to audiotactile asynchrony. Neurosci. Lett. 413, 72–76 10.1016/j.neulet.2006.11.027 17161530

[B33] Navarra J. Vatakis A. Zampini M. Soto-Faraco S. Humphreys W. Spence C. (2005). Exposure to asynchronous audiovisual speech extends the temporal window for audiovisual integration. Brain Res. Cogn. Brain Res. 25, 499–507 10.1016/j.cogbrainres.2005.07.009 16137867

[B34] Okada M. Kashino M. (2003). The role of spectral change detectors in temporal order judgment of tones. Neuroreport 14, 261–264 10.1097/00001756-200310060-00002 12598742

[B35] Parise C. Spence C. (2009). ‘When birds of a feather flock together’: synesthetic correspondences modulate audiovisual integration in nonsynesthetes. PLoS ONE 4:e5664 10.1371/journal.pone.0005664 19471644 PMC2680950

[B36] Parise C. V. Spence C. Ernst M. O. (2012). When correlation implies causation in multisensory integration. Curr. Biol. 22, 46–49 10.1016/j.cub.2011.11.039 22177899

[B37] Roach N. W. Heron J. Whitaker D. McGraw P. V. (2011). Asynchrony adaptation reveals neural population code for audio-visual timing. Proc. R. Soc. Lond. B Biol. Sci. 278, 1314–1322 10.1098/rspb.2010.1737 PMC3061136 20961905

[B38] Roseboom W. Arnold D. H. (2011). Twice upon a time: multiple, concurrent, temporal recalibrations of audio-visual speech. Psychol. Sci. 22, 72–877 10.1177/0956797611413293 21690312

[B39] Roseboom W. Kawabe T. Nishida S. (2013). Direction of visual apparent motion driven by perceptual organization of cross-modal signals. J. Vis. 13, 1–13 10.1167/13.3.1 23291646

[B40] Roseboom W. Nishida S. Arnold D. H. (2009). The sliding window of audio-visual simultaneity. J. Vis. 9, 1–8 10.1167/9.13.1 20053095

[B41] Roufs J. A. (1963). Perception lag as a function of stimulus luminance. Vision Res. 3, 81–91 10.1016/0042-6989(63)90070-1

[B42] Spence C. Deroy O. (2013). How automatic are crossmodal correspondences? Conscious. Cogn. 22, 245–260 10.1016/j.concog.2012.12.006 23370382

[B43] Spence C. Shore D. I. Klein R. M. (2001). Multisensory prior entry. J. Exp. Psychol. Gen. 130, 799–832 10.1037/0096-3445.130.4.799 11757881

[B44] Spence C. Squire S. B. (2003). Multisensory integration: maintaining the perception of synchrony. Curr. Biol. 13, R519–R521 10.1016/S0959-440X(03)00103-9 12842029

[B45] Stein B. E. Meredith M. A. (1993). The Merging of the Senses. Cambridge, MA: MIT Press

[B46] Tanaka A. Kaori A. Hisato I. (2011). The change in perceptual synchrony between auditory and visual speech after exposure to asynchronous speech. Neuroreport 22, 684–688 10.1097/WNR.0b013e32834a2724 21817926

[B47] Titchener E. B. (1908). Lecture on the Elementary Psychology of Feeling and Attention. New York: Macmillan

[B48] van Eijk R. L. J. Kohlrausch A. Juola J. F. van de Par S. (2008). Audiovisual synchrony and temporal order judgments: effects of experimental method and stimulus type. Percept. Psychophys. 70, 955–968 10.3758/PP.70.6.955 18717383

[B49] Vatakis A. Navarra J. Soto-Faraco S. Spence C. (2007). Temporal recalibration during asynchronous audiovisual speech perception. Exp. Brain Res. 181, 173–181 10.1007/s00221-007-0918-z 17431598

[B50] Vatakis A. Navarra J. Soto-Faraco S. Spence C. (2008). Audiovisual temporal adaptation of speech: temporal order versus simultaneity judgments. Exp. Brain Res. 185, 521–529 10.1007/s00221-007-1168-9 17962929

[B51] Vatakis A. Spence C. (2006). Audiovisual synchrony perception for music, speech, and object actions. Brain Res. 1111, 134–142 10.1016/j.brainres.2006.05.078 16876772

[B52] Vatakis A. Spence C. (2007). Crossmodal binding: evaluating the “unity assumption” using audiovisual speech stimuli. Percept. Psychophys. 69, 744–756 10.3758/BF03193776 17929697

[B53] Vatakis A. Spence C. (2008). Evaluating the influence of the ‘unity assumption’ on the temporal perception of realistic audiovisual stimuli. Acta Psychol. (Amst.) 127, 12–23 10.1016/j.actpsy.2006.12.002 17258164

[B54] Vroomen J. Keetels M. (2010). Perception of intersensory synchrony: a tutorial review. Atten. Percept. Psychophys. 72, 871–884 10.3758/APP.72.4.871 20436185

[B55] Vroomen J. Keetels M. de Gelder B. Bertelson P. (2004). Recalibration of temporal order perception by exposure to audio-visual asynchrony. Cogn. Brain Res. 22, 32–35 10.1016/j.cogbrainres.2004.07.003 15561498

[B56] Williams J. M. Lit A. (1983). Luminance-dependent visual latency for the Hess effect, the Pulfrich effect, and simple reaction time. Vision Res. 23, 171–179 10.1016/0042-6989(83)90140-2 6868392

[B57] Yamamoto S. Miyazaki M. Iwano T. Kitazawa S. (2012). Bayesian calibration of simultaneity in audiovisual temporal order judgments. PLoS ONE 7:e40379 10.1371/journal.pone.0040379 22792297 PMC3392227

[B58] Yarrow K. Roseboom W. Arnold D. H. (2011a). Spatial grouping resolves ambiguity to drive temporal recalibration. J. Exp. Psychol. Hum. Percept. Perform. 37, 1657–1661 10.1037/a0024235 21688937

[B59] Yarrow K. Jahn N. Durant S. Arnold D. H. (2011b). Shifts of criteria or neural timing? The assumptions underlying timing perception studies. Conscious. Cogn. 20, 1518–1531 10.1016/j.concog.2011.07.003 21807537

[B60] Yuan X. Li B. Bi C. Yin H. Huang X. (2012). Audiovisual temporal recalibration: space-based versus context-based. Perception 41, 1218–1233 10.1068/p7243 23469702

